# Supplementary material for: Comparative genomics provides new insights into the diversity, physiology, and sexuality of the only industrially exploited tremellomycete: Phaffia rhodozyma
Source: BMC Genomics. 2016 Nov 9;17:901. doi: 10.1186/s12864-016-3244-7 (PMC5103461; doi:10.1186/s12864-016-3244-7)
Supplement: Additional file 6: — List of orphan genes with links to PFAM (related to Additional file 1: Table S1). (ZIP 1428 kb) [file 12864_2016_3244_MOESM6_ESM.zip › PFAM_UNIQUE/G03036_P.html]

#                                                                            --- full sequence --- -------------- this domain -------------   hmm coord   ali coord   env coord
  
# target name        accession   tlen query name           accession   qlen   E-value  score  bias   #  of  c-Evalue  i-Evalue  score  bias  from    to  from    to  from    to  acc description of target
  
#------------------- ---------- ----- -------------------- ---------- ----- --------- ------ ----- --- --- --------- --------- ------ ----- ----- ----- ----- ----- ----- ----- ---- ---------------------
  
G03036\_P             -            757 ADIP                 PF11559.3    151       4.8    5.7  13.1   1   2       7.9   2.9e+03   -3.3   1.0    69   100   553   584   539   591 0.48 - Afadin- and alpha -actinin-Binding  
G03036\_P             -            757 ADIP                 PF11559.3    151       4.8    5.7  13.1   2   2   0.00022     0.083   11.5   5.2    57   138   629   710   623   721 0.84 - Afadin- and alpha -actinin-Binding  
G03036\_P             -            757 Atg14                PF10186.4    302      0.13    9.9   3.4   1   1    0.0012      0.27    8.8   3.4    53   131   616   699   546   723 0.59 - UV radiation resistance protein and autophagy-related subunit 14  
G03036\_P             -            757 Cast                 PF10174.4    775     0.024   11.4   8.6   1   1   0.00019     0.041   10.6   8.6   227   384   557   730   521   753 0.64 - RIM-binding protein of the cytomatrix active zone  
G03036\_P             -            757 CENP-F\_leu\_zip       PF10473.4    140         3    6.4  19.8   1   3       9.4   1.8e+03   -2.6   1.3    94   101   574   581   540   595 0.56 - Leucine-rich repeats of kinetochore protein Cenp-F/LEK1  
G03036\_P             -            757 CENP-F\_leu\_zip       PF10473.4    140         3    6.4  19.8   2   3   3.7e-05    0.0072   14.9   8.0    32   122   618   709   614   727 0.85 - Leucine-rich repeats of kinetochore protein Cenp-F/LEK1  
G03036\_P             -            757 CENP-F\_leu\_zip       PF10473.4    140         3    6.4  19.8   3   3         3   5.8e+02   -1.0   0.1     9    33   726   750   715   752 0.78 - Leucine-rich repeats of kinetochore protein Cenp-F/LEK1  
G03036\_P             -            757 DivIVA               PF05103.8    131       3.2    6.5  10.4   1   2       3.3   1.3e+03   -2.0   0.4    46    59   574   587   540   599 0.55 - DivIVA protein  
G03036\_P             -            757 DivIVA               PF05103.8    131       3.2    6.5  10.4   2   2   0.00057      0.23   10.2   4.0    35   105   641   714   609   736 0.69 - DivIVA protein  
G03036\_P             -            757 DUF1664              PF07889.7    126    0.0053   15.2   4.0   1   2     0.045        16    4.0   0.2    65    99   547   581   536   594 0.80 - Protein of unknown function (DUF1664)  
G03036\_P             -            757 DUF1664              PF07889.7    126    0.0053   15.2   4.0   2   2   0.00015     0.053   12.0   0.4    49   124   611   687   608   689 0.87 - Protein of unknown function (DUF1664)  
G03036\_P             -            757 DUF2353              PF09789.4    319    0.0014   16.6   4.1   1   1   4.6e-06    0.0023   15.9   4.1    47   165   615   735   560   746 0.77 - Uncharacterized coiled-coil protein (DUF2353)  
G03036\_P             -            757 DUF972               PF06156.8    107       1.7    7.7   7.7   1   2       4.9   1.1e+03   -1.4   0.0    34    55   622   643   614   651 0.71 - Protein of unknown function (DUF972)  
G03036\_P             -            757 DUF972               PF06156.8    107       1.7    7.7   7.7   2   2   0.00053      0.12   11.4   1.2     6    70   650   714   647   730 0.79 - Protein of unknown function (DUF972)  
G03036\_P             -            757 Nup54                PF13874.1    141      0.35    9.2   3.0   1   1    0.0014       1.2    7.4   3.0    43   134   630   716   628   725 0.77 - Nucleoporin complex subunit 54  
G03036\_P             -            757 Snapin\_Pallidin      PF14712.1     92       6.1    5.9   9.3   1   2     0.011       5.5    6.1   2.2    15    52   547   584   544   594 0.89 - Snapin/Pallidin  
G03036\_P             -            757 Snapin\_Pallidin      PF14712.1     92       6.1    5.9   9.3   2   2     0.083        41    3.3   0.7    12    81   636   676   618   687 0.61 - Snapin/Pallidin  
G03036\_P             -            757 Spc7                 PF08317.6    326       2.2    5.5   5.2   1   1     0.024       4.4    4.5   4.8   207   257   629   679   542   705 0.64 - Spc7 kinetochore protein  
G03036\_P             -            757 Syntaphilin          PF15290.1    305       8.2    4.5  10.3   1   2      0.24   7.3e+02   -1.9   4.1   190   283     3    97     1   154 0.64 - Golgi-localised syntaxin-1-binding clamp  
G03036\_P             -            757 Syntaphilin          PF15290.1    305       8.2    4.5  10.3   2   2   0.00013      0.39    8.8   0.4    69   137   646   709   640   715 0.92 - Golgi-localised syntaxin-1-binding clamp  
G03036\_P             -            757 TBPIP                PF07106.8    169       0.1   10.8   4.3   1   1    0.0007      0.26    9.5   4.3    70   165   622   719   615   723 0.75 - Tat binding protein 1(TBP-1)-interacting protein (TBPIP)
